# Enhanced isolation in aperture fed dielectric resonator MIMO antennas for 5G Sub 6 GHz applications

**DOI:** 10.1038/s41598-025-95040-8

**Published:** 2025-03-27

**Authors:** Arpita Patel, Trushit Upadhyaya, Pandey Rajat Girjashankar, M. V. Swati, Om Prakash Kumar

**Affiliations:** 1 Electronics and Communication Department, Chandubhai S. Patel Institute of Technology, Charotar University of Science & Technology, Changa, Gujarat India; 2 Electronics and Communication Department, Government Engineering College, Gandhinagar, Gujarat India; 3 Department of Electronics and Communications Engineering, National Institute of Technology (NIT) Silchar, Silchar, Assam 788010 India; 4 Department of Electronics and Communication Engineering, Manipal Institute of Technology, Manipal Academy of Higher Education, Manipal, 576104 India

**Keywords:** Dielectric resonator antenna (DRA), MIMO antenna, 5G Sub-6 GHz, Rectangular dielectric resonators, 5G technology, SDG 9, Electrical and electronic engineering, Engineering

## Abstract

A quad-port dielectric resonator antenna (DRA) is proposed for sub-6 GHz 5G MIMO applications, featuring high isolation, dual-band operation, and enhanced efficiency. The antenna is designed using electromagnetic coupling with a triangular patch to excite the square dielectric resonators (DRs) at targeted resonance modes (TE^x^_111_ and TE^x^_121_), achieving broadband and polarization diversity. The proposed structure exhibits self-isolation above 20 dB without requiring additional decoupling structures. The use of Alumina (εr = 9.9, tanδ = 0.0001) ensures low loss, high efficiency (88.9% and 93.8%), and strong radiation performance, with peak gains of 9.12 dBi and 8.58 dBi at 3.72 GHz and 4.75 GHz, respectively. The full ground plane and optimized spatial placement further contribute to reduced mutual coupling and improved diversity performance, achieving an envelope correlation coefficient (ECC) of 0.042 and channel capacity loss (CCL) below 0.2 bits/s/Hz. The proposed antenna’s measured results align closely with simulations, confirming its suitability for high-performance 5G MIMO communication systems.

## Introduction

Fast communication is a progressive requirement for current-generation technologies. Existent 3G/4G communication systems do not meet ultra-high-speed streaming requirements. Multiple-input, multiple-output technologies easily fulfil LTE, LTE Advanced, and 5G communication necessities. These systems provide the ability to improve channel capacity by employing multiple transmitters and receivers in the communication system. The objectives of the present study are: (1) To design a MIMO antenna for high-data-rate communication. (2) The designed antenna shall present dual-band resonance covering the 5G sub-6 GHz communication. The 5th generation communication system is capable of providing extremely low latency and high data rate^1^. However, co-channel interference is a vital difficulty while multiple user allocation occurs within the spectrum. The non-line-of-sight scenarios pose hostile challenges for desired communication attributes, viz., low bit error rate, QoS, and spectral efficiency, to name a few. The advancement of every new generation results in considerable enhancement of data rates. The increase occurs given the capabilities of high spectrum efficiency in 5G communication systems. The increase in transmission rate and signal strength leads to the marginal tradeoff in the bandwidth and transmission power, however, there is a definite increase in system complexity and footprint on board. The miniaturization of such antennas shall come with the cost of inter-element isolation due to the mutual coupling of closed proximity elements.

The enhanced communication reliability in 5G communication occurs due to the inclusion of multiple antennas on board. The multi-antenna system is associated with distinct and spatially separated antenna elements, which sometimes exhibit different polarizations. By means of multiple onboard resonators, parallel isolated wireless communication channels can be perceived in a multipath environment, causing an increase in the overall channel capacity. Due to the extremely space-restraint environment of antennas within the terminal modules, it is very important to optimize the dimensions of the antennas. Surface mountable antennas have become an obvious choice for addressing this limitation^2,3^. The patch antennas are an obvious selection for cost-effective mass production. However, they ominously suffer from enormously severe dielectric losses. Dielectric resonator antennas (DRAs) offer high radiation efficiency at microwave and millimeter-wave frequencies due to their low conduction losses, making them a strong alternative to traditional patch antennas, particularly for high-frequency applications. The adequate electrical dimensions, typically in proportion to wavelength, shall possess a lower form factor. DRAs also possess larger impedance bandwidth and gain with an appropriate choice of the dielectric constant in contrast to patch antenna^4^. By altering the electrical length comparable to its wavelength, the antenna parameters can be finely tuned. The literature avails MIMO antenna research in substantial quantum. There are certain critical issues with MIMO antenna technologies targeting base station applications in sub-6 GHz band applications^5^. It presents an 11 × 11 MIMO antenna array of multimode elements for the implementation of MIMO antenna attaining high diversity parameters. In another design, two port MIMO antennae having L-shaped tuned stubs optimized for T-shaped monopole slots in the ground plane are presented^6^. It also analyzes specific absorption rates for human hands using a suggested MIMO antenna. A comprehensive review of MIMO antenna utilized in various systems for wireless communication is available in the literature^7^. It covers a wide spectrum of MIMO antennae, covering the challenges and tradeoffs of antenna compactness against the MIMO antenna diversity parameters for 5G applications. A high bandwidth MIMO antenna for 5G smartphone applications is exhibited in^8^. A patch with a Reclined-F slot for as high as 88% bandwidth is proposed for eight antenna elements. A sub-6 GHz along with mm-wave 5G-v2x MIMO antenna array with tapering slit antenna is implemented along with a dipole to cover both frequency bands with 360-degree coverage^9^. A low-profile 5G MIMO antenna for the NR-n2 band is proposed for large-scale applications^10^. The compact design incorporates ring and loop slots to achieve the targeted resonance. A multilayer massive MIMO antenna array system loaded with metamaterials. Each layer consists of multiple patches to achieve a small volume-based MIMO array system^11^. The detailed perspective of self-isolated 5G FR-2 band application antennas has been exhibited where complementary split ring resonators are employed for achieving the wideband resonance in the FR-2 band^12^.

However, the existing literature emphasizing the DRA MIMO antenna for 5G communications. Circularly polarized two-layered dielectric resonator antennas have been proposed in^13^. The design provides RHCP with a fractional bandwidth in the order of 9% for sub-6 GHz communication applications. The mm-wave MIMO antenna through rDRA and cDRA, wherein the DRA were manufactured using 3D printing technology, attains a very high gain of 6.6 dBi and 12 dBi for achieved dual bands^14^. A CPW-fed DRA for 5G NR bands wherein the symmetric cross-shape DRAs are utilized for dual orthogonal modes being excited for achieving pattern diversity for MIMO applications^15^. The 4-element rectangular DRA with an extremely small electrical footprint with antenna gain & radiation efficiency is shown in^16^. The mutual coupling reduction in closely shaped DRA with extremely high efficiency in order of 93% is exhibited^17^. Conductive metallic strips are utilized to isolate the DRA to achieve high spectral diversity. A good combination of DRA, electromagnetic bandgap, and DGS structure is utilized in C-band communication applications. The design has 8 × 8 MIMO elements for achieving high efficiency^18^. A CPW-fed quad-port DRA for NR N47 sub-6 GHz applications wherein a cross-shaped structure is tuned for high efficiency for achieving wideband in order of 11% for dual-band resonance^19^.

The hybrid DRA, which is fed by a meandered printed line, is presented, wherein the electrical dimensions are very compact, and the antenna provides an extraordinary gain in the order of 13 dBi for the Sub-6 GHz spectrum^20^. The design provides fair pattern diversity. A two-dielectric layers-based DRA is given, wherein, due to multiple dielectric layers, an extremely high bandwidth is achieved^13^. Furthermore, owing to the presented design, a circular polarization was also achieved along with high port isolation. Aperture-fed DR aimed at sub-6 GHz & WLAN application is accessible in^21^. Due to the sparse orientation of conducting elements and owing to design optimization, good polarization, and spatial diversity have been presented. In additional design, a quad-port DRA for sub-6 GHz and WiMAX communications is presented^22^. The DRA had two operating modes, TE01δ and TE10δ, giving fair gain and substantial bandwidth. A tunable MIMO DRA antenna was presented, whereas a circular-type DRA was presented. The mechanical tuning provides switching between frequencies by rotating the antenna slots. This antenna gives an extremely high fractional bandwidth in the order of 20%^23^. The modified ground-based enhanced isolation is presented at sub-6 GHz frequency applications of multiple countries^24^. The T-formed structure is kept underground to provide isolation. The antenna has fair bandwidth and diversity parameters. Metasurface-inspired MIMO DRA has been presented; the electrically small and highly isolated antenna was able to provide its application for the n-77 band^25^. In a nutshell, the dielectric resonator’s electrical dimensions and structure are extremely vital in the mode being excited for antenna resonance. The inter-element positioning primarily affects the isolation between the ports. This isolation is a critical factor in achieving diversity. The port isolation enhancement can be done through varied techniques as available in the wide literature^26–29^. The easiest way to improve port isolation is through a sparse arrangement of the resonating elements. The key tradeoff between miniaturization and isolation must be addressed throughout the design of the MIMO array; this shall eventually balance commercial production versus MIMO performance parameters. This yields major design challenges for the antenna designer. It is very commonly observed to have a larger gain and bandwidth for dielectric-inspired antennas. The improvement in the cross-polarization is also possible with the induction of dielectric material. Mode decoupling is one of the ways to reduce isolation constraints. The optimal positioning of the dielectric resonators alters the current directions, causing a significant reduction in inter-element coupling. A linearly polarized MIMO DRA antenna is designed for WLAN applications^30^. The design presents a multi-element bowtie design with high fractional bandwidth. In another design of linearly polarized MIMO DRA, capacitive loaded loops are incorporated to enhance the isolation and, subsequently, diversity parameters of the MIMO antenna^31^. A composite feeding structure was presented to achieve triple radiating modes in cylindrical DRA^32^. The design has excited DRA for a couple of hybrid modes and one TM mode for the excitation of three distinct resonances. A circular dielectric-based MIMO design is presented wherein high space and polarization diversity is achieved^33^. The dual hybrid modes were excited using the circular dielectric material. Despite significant advancements in MIMO antenna technology, several challenges remain unaddressed for 5G sub-6 GHz applications. Most MIMO antennas suffer from high mutual coupling, necessitating additional decoupling techniques such as electromagnetic bandgap (EBG) structures or defected ground structures (DGS), which increase design complexity and fabrication cost^25,27,29^. Additionally, while dielectric resonator antennas (DRAs) offer reduced conduction losses, many existing designs focus on single-band operation, limiting their applicability for 5G networks that require multi-band support^20–22^. Furthermore, existing MIMO DRA designs often achieve low gain (< 7 dBi) and moderate efficiency (~ 80%) due to suboptimal coupling and radiation losses^13,18,22^.

This work presents a quad-port dielectric resonator MIMO antenna that achieves high isolation (> 20 dB) without the need for additional decoupling structures. It operates at dual bands of 3.72 GHz and 4.75 GHz, delivering improved gain (9.12 dBi and 8.58 dBi) and high efficiency (88.9% and 93.8%). With wide bandwidths of 14.7% and 11.57%, the antenna ensures reliable communication, making it well-suited for 5G Sub-6 GHz applications, particularly in the n77, n78, and n79 bands. The novelty of the designed dielectric resonator antenna (DRA) lies in its ability to excite the TE^x^_111_ and TE^X^_121_ modes while achieving the targeted resonance frequencies. The proposed work offers distinct technical advantages over existing designs, including:

1. Enhanced isolation without additional structures—The design achieves high isolation (> 20 dB) solely through strategic spatial placement of dielectric resonators and orthogonal polarization, eliminating the need for electromagnetic bandgap (EBG) structures, parasitic elements, or neutralization lines commonly used in sub-6 GHz MIMO antennas.
2. Dual-band operation with higher-order DR modes—By exciting higher-order dielectric resonator (DR) modes, the antenna achieves improved bandwidth (14.7% and 11.57% at the two resonance frequencies), surpassing traditional patch-based MIMO antennas.
3. Superior efficiency and gain—The use of alumina (εr = 9.9, tanδ = 0.0001) minimizes conduction losses, leading to high radiation efficiency (88.9% & 93.8%) and peak gain (9.12 dBi & 8.58 dBi).
4. Simplified fabrication and scalability—Unlike conventional designs that require intricate isolation techniques, this design maintains a full ground plane for ease of fabrication while ensuring scalability to larger MIMO arrays.
5. Optimized for 5G Sub-6 GHz applications—The proposed antenna meets key MIMO performance metrics, including an envelope correlation coefficient (ECC) of 0.042, a Channel Capacity Loss (CCL) below 0.2 bits/s/Hz, and a Diversity Gain (DG) of 9.99, making it well-suited for next-generation wireless communications.
  These innovations collectively enhance the reliability, efficiency, and manufacturability of MIMO antennas for sub-6 GHz 5G applications. The following sections provide a detailed sensitivity analysis, MIMO performance evaluation, and prototype validation.

## Antenna design configuration

**Fig. 1:**
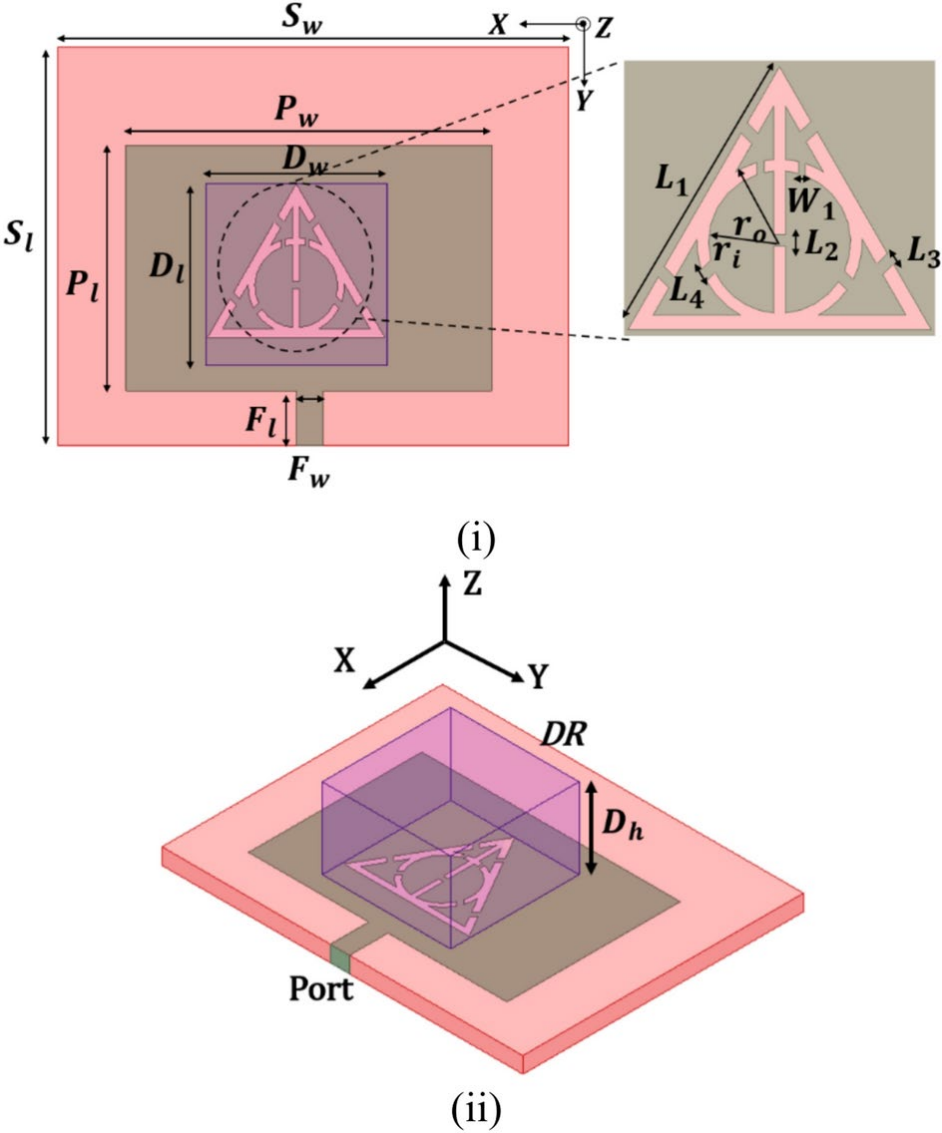
DR antenna (**i**) Top (**ii**) 3D.

The designed dielectric resonator (DR) antenna is illustrated in Fig. 1, showing both the top and three-dimensional views for clear identification. The initial design aimed to produce a single resonance by exciting the patch in the TM10 mode, which has a minimal electric field at the center. To achieve dual-band operation, an additional resonance was introduced by exciting the dielectric resonator at targeted frequencies. While a patch is used for excitation, the majority of the radiation comes from the dielectric resonator, which helps achieve efficient radiation and lower metallic losses compared to conventional patch antennas at one of the targeted resonances

The DR antenna can excite various modes by adjusting its electrical dimensions, leading to high efficiency and a compact form factor. This versatility is enhanced by using high-dielectric-constant materials, such as alumina with a dielectric constant of 9.9 and a loss tangent of 0.0001. These properties ensure high bandwidth and efficiency even with a high Q-factor. Tuning across a wide frequency range is possible by selecting materials with different permittivity values.

High-permittivity materials allow a significant reduction in the antenna’s electrical size without increasing its physical footprint. DRAs support multiple resonant modes, enhancing bandwidth compared to conventional antennas. The high permittivity concentrates more energy within the structure, resulting in higher gain, while the low-loss properties further improve performance. The ability to adjust the size and shape of the resonator allows for tailored radiation patterns, greater directivity, and stable high gain across the bandwidth.

Alumina maintains consistent dielectric properties across a wide temperature range, ensuring reliable performance under different thermal conditions. Its hydrophobic nature prevents moisture absorption, making it suitable for outdoor and high-humidity environments. Although specific stability testing was not conducted, alumina’s proven reliability in other applications supports its use in diverse conditions.

The proposed design uses a Dielectric Resonator Antenna (DRA) to improve efficiency, bandwidth, and gain. By using Alumina-based DRAs, the antenna achieves better performance compared to conventional materials, making it a great choice for compact, high-efficiency, and reliable 5G MIMO applications. This material helps ensure strong isolation, higher gain, and stable operation, effectively addressing key challenges in sub-6 GHz wireless communication.

There are multiple ways to excite the DR-based antenna, such as an electromagnetically, Microstrip, aperture feed, and so on. The presented design is electromagnetically coupled to feed the DRA. This technique was selected because of its ease of design and manufacturing process. The electromagnetic coupling felicitates the excitation of DR through an electromagnetic coupling of the field being emitted by the resonating patch. The coupled field gets stored by DR based on the electric properties of the dielectric material. The field in dielectric material is stored through the re-orientation of the irregularly aligned dipoles, and thus, DR gets excited to defined modes.

The ${TE}_{111}^{x}$ mode is the fundamental transverse electric mode in the DRA, where the field exhibits a single half-wavelength variation along each spatial axis. This mode is primarily responsible for broadside radiation and efficient energy confinement within the dielectric structure, making it suitable for applications requiring high radiation efficiency and stable impedance characteristics. The ${TE}_{121}^{X}$ mode is a higher-order mode that introduces additional field variations along the resonator, leading to enhanced radiation directivity and potential bandwidth improvement. The excitation of this mode through a patch provides stronger coupling, making it beneficial for advanced antenna designs requiring higher gain and multi-frequency operation. These modes are crucial in shaping the DRA’s overall performance, influencing parameters such as gain, bandwidth, polarization, and radiation efficiency.

**Fig. 2:**
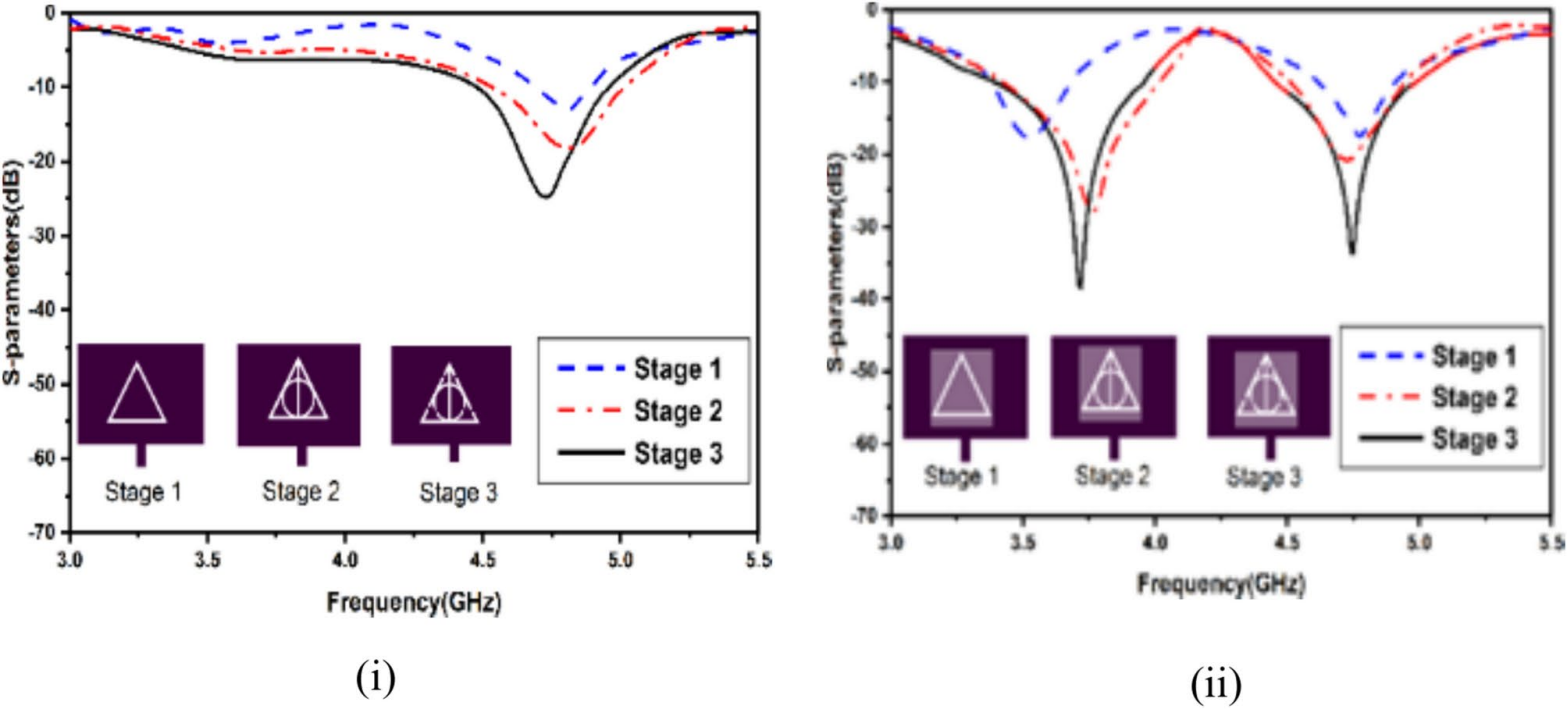
Phase-wise electromagnetically feed development (**i**) without DR (**ii**) with DR.

The optimized dimensions of the patch, DR, and slots were selected for exciting the DRA at ${TE}_{111}^{x} \text{and} {TE}_{121}^{X}$ modes which is a novel attribute of the presented design. Figure 2 illustrates the process of optimizing the patch slots to enhance the coupling for improved antenna radiation parameters. The phase-wise evolution of the antenna with and without the DR element is shown. The choice of a triangular-shaped slot was based on extensive simulations and optimization to achieve efficient coupling, impedance matching, and mode excitation in the dielectric resonator. The choice was done after numerous iterations of the slots over other shapes available in literature. One of the technical justification which we think is that the triangular slot allows a more uniform and efficient coupling of electromagnetic fields into the dielectric resonator, leading to improved excitation of the desired resonant modes.

The mechanical dimensions of the antenna are mentioned in Table 1. The antenna is manufactured on an FR-4 substrate having copper sheets worth a thickness of 70 μm on either side of the substrate for etching the design. The proposed DR has an equal length and width of 16 mm and a height of 10mm. The square DR is usually preferred over the circular or cylindrical DR due to ease and feasibility in design optimization. In addition, the degenerative modes are being evaded by rectangular DR. It is extremely difficult to carry out the prediction of the combined Patch and DR-based resonator for the tailoring of the frequencies; however, through inbuilt support of software simulator design has been optimized to resonate covering designated applications. The tailored electrical dimensions of the rectangular DR were extracted through design equations for the generation of the fundamental mode. The calculated values for the first & subsequent resonant frequencies were 3.78 GHz & 4.80 GHz, respectively, whereas the simulated values were 3.72 GHz and 4.75 GHz.

**Table 1:** Geometrical parameters of a single-element antenna.

Parameter	Dimensions(mm)	Parameter	Dimensions(mm)
Pw (Width of patch)	32.23	Ws (Substrate width)	90
P_l_ (Length of patch)	21.6	Ls (Substrate length)	90
F_W_ (Width of feed)	2.4	W_1_(width between inner and outer circle)	0.37
F_l_ (Length of feed)	4.8	L_3_ (Slit of L_1_)	0.62
D_W_ (Width of DRA)	16	L_5_ (Diameter of outer line)	0.62
D_l_ (Length of DRA)	16	D_h_ (Height of DRA)	10

The proposed dielectric resonator antenna (DRA) is designed to operate within the 5G Sub-6 GHz frequency range, covering 3.72 GHz and 4.75 GHz as its primary resonance frequencies. These frequencies fall within the sub-6 GHz spectrum allocated for 5G communications, which includes bands such as n77 (3.3–4.2 GHz), n78 (3.3–3.8 GHz), and n79 (4.4–5.0 GHz).

It can be witnessed that there is a close approximation of computed and simulated resonances. A slight discrepancy arises since a dielectric waveguide design does not completely accommodate an electromagnetically coupled feeding mechanism.

Upon solving the fields inside the DR of the antenna in a simulator, excitation of fundamental mode can be perceived utilizing E-Field within DR. Figure 4 depicts the mode excitation at ${TE}_{111}^{x}$ and ${TE}_{121}^{X}$. The E-field distribution in the x–z and y–z dimensions of the DR exhibits generation of the higher order modes. The mode calculations were carried out by the dielectric waveguide model technique as follow^20^:

1 $${k}_{z}tan\left(\frac{{k}_{z}h}{2}\right)=\sqrt{\left({\varepsilon }_{r}-1\right)\left({{k}_{o}}^{2}\right)-{{k}_{z}}^{2}}$$

2 $${{k}_{x}}^{2}+{{k}_{y}}^{2}+{{k}_{z}}^{2}={\varepsilon }_{r}{{k}_{o}}^{2}$$

3 $$k_{x} = \frac{m\pi }{l};\;k_{y} = \frac{n\pi }{w};\;k_{o} = \frac{2\pi }{{\lambda_{o} }}$$

${k}_{o}$: free-space wavenumber; ${k}_{x}$,${k}_{y},$ and ${k}_{z}$: λ/2 variations; *l, w* and *h*: DR length, width, and height.

**Fig. 3:**
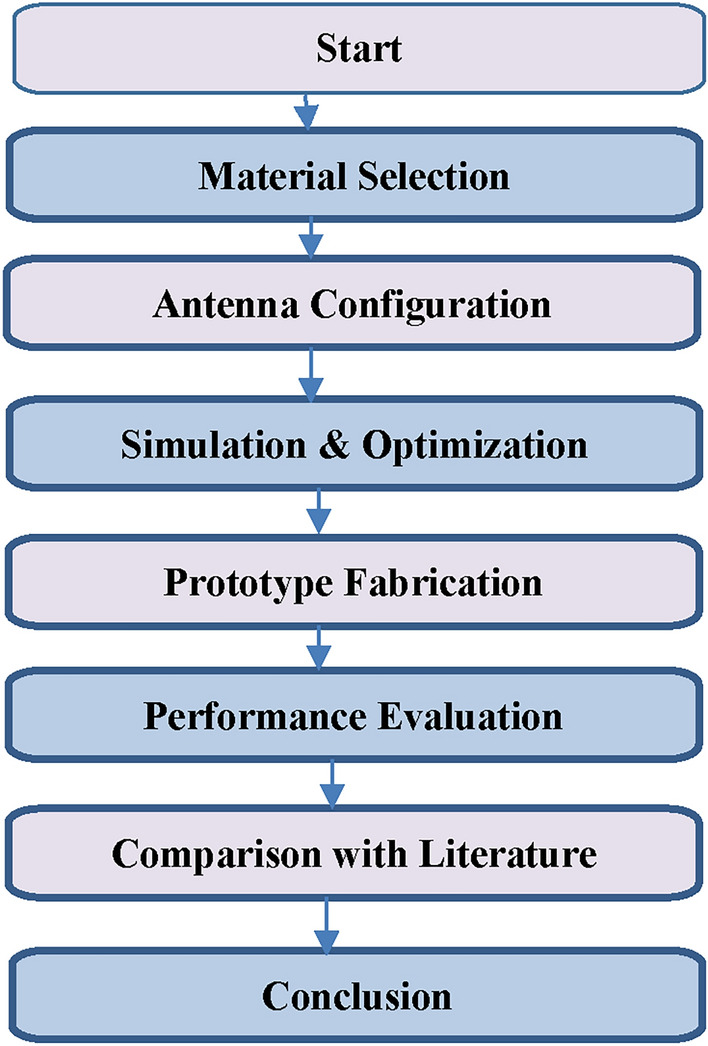
Methodology of the proposed design.

Figure 3 illustrates the methodology adopted in the design of the proposed dielectric resonator MIMO antenna. The process begins with the selection of appropriate dielectric materials and resonator dimensions, followed by mode excitation through electromagnetic coupling. The antenna design is then optimized using simulations to achieve high isolation and dual-band operation. Finally, performance evaluations are carried out to validate key parameters such as gain, efficiency, and isolation, ensuring suitability for 5G Sub-6 GHz applications.

**Fig. 4:**
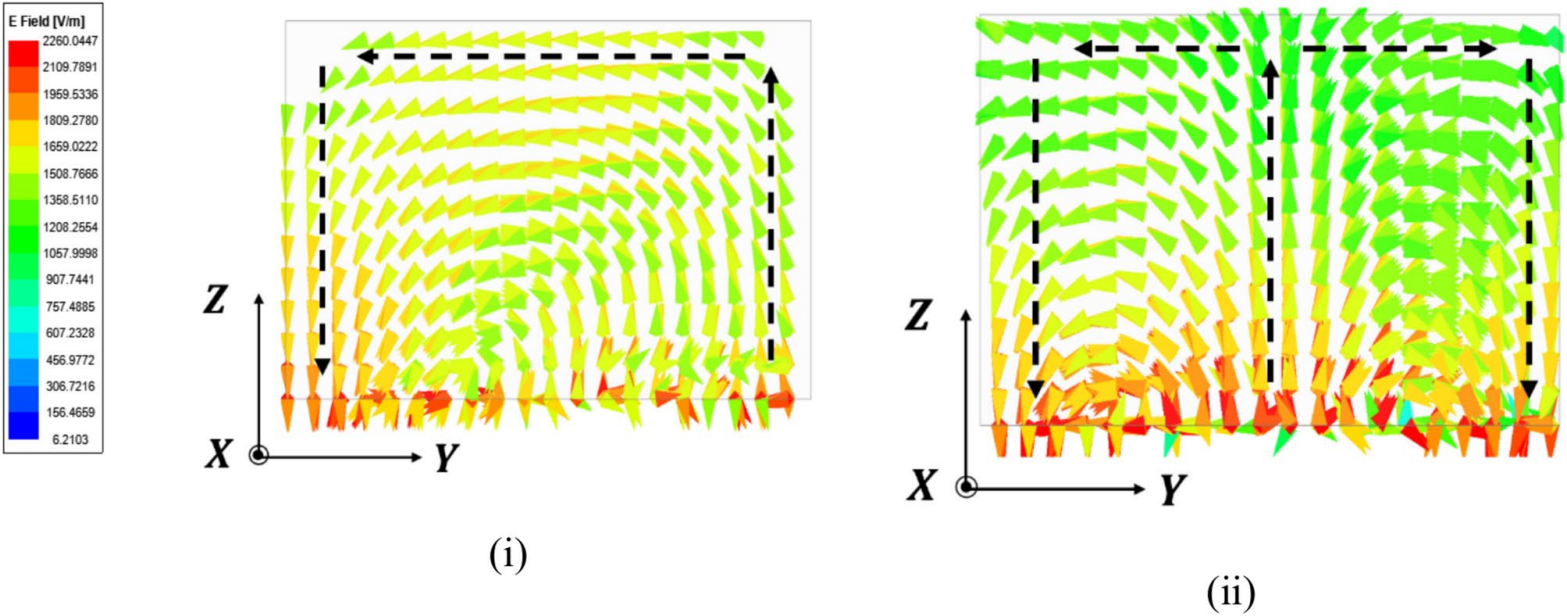
Excited E-field (**i**) ${TE}_{111}^{X}$ (**ii**) ${TE}_{121}^{X}$.

The design methodology of the novel four-element rectangular dielectric resonator antenna focuses on selecting specific modes to achieve optimal performance, spatial and polarization diversity, and high isolation. Each DRA element measures 16 × 16 × 10 mm^3^ and is composed of alumina, exhibiting an impressive dielectric factor of 9.9 and an exceptionally small loss tangent around 0.0001. This material selection maximizes compactness and performance, guaranteeing effective radiation efficiency. An innovative electromagnetic coupling is employed to stimulate the dielectric resonator via electromagnetic coupling, enhancing energy transfer efficiency. The connection holes are meticulously tuned to stimulate the designated mode within the DRAs. The selected modes ${TE}_{111}^{x}$ and ${TE}_{121}^{X}$ are crucial for achieving the desired performance. The ${TE}_{111}^{x}$ and ${TE}_{121}^{X}$ mode offers broadside radiation with good impedance bandwidth. This mode corresponds to the fundamental mode in a rectangular DRA, where the electric field varies half-wave along the length, width, and height, ensuring a stable radiation pattern across a wide frequency range. The ${TE}_{111}^{x}$ and ${TE}_{121}^{X}$ modes, chosen for polarization diversity, have a half-wave variation along the length and height and a full-wave variation along the width. Exciting this mode orthogonally to the ${TE}_{111}^{x}$ and ${TE}_{121}^{X}$ mode allows the antenna to achieve polarization diversity, which helps mitigate multipath fading and improve signal quality in MIMO systems (Fig. 4).

Spatial diversity & polarization diversity is utilized by configuring the four-element arrangement to minimize mutual coupling and maximize isolation, attaining isolation above 21 dB over its operational frequency range. The dimensions and configuration of the dielectric resonator significantly impact the isolation and coupling of the MIMO antenna, influencing its performance from multiple perspectives. Mutual coupling refers to the field interaction between the MIMO antenna elements, while isolation reduces this mutual coupling, improving the overall performance. The dimensions of the dielectric resonator antenna (DRA) play a crucial role, as larger DRAs tend to exhibit stronger near-field interactions, increasing coupling. Conversely, smaller DRAs reduce coupling but may compromise the advantages of using a dielectric resonator, such as gain and bandwidth. The spacing between DR elements is equally vital, as greater inter-element spacing lowers coupling but can negatively affect spatial diversity. Furthermore, the geometry of the resonators also affects coupling parameters. Cylindrical resonators, although simple in structure, demonstrate significant mutual coupling when in close proximity. Rectangular resonators provide better coupling responses compared to cylindrical ones but may create stronger interactions with adjacent elements due to their directional radiation pattern. Hemispherical resonators, being compact, offer superior isolation when adequately placed.

The feed mechanism and placement relative to the DRA dimensions also significantly impact mutual coupling. Poor feed placement can drastically reduce isolation in MIMO antennas, while Microstrip lines are known to suppress mutual coupling effectively. A well-designed combination of DRA dimensions and arrangement can achieve high isolation, reduced mutual coupling, and optimized MIMO system performance. In the proposed design, isolation is achieved through three primary mechanisms. First, the use of orthogonal modes, specifically the TE^X^_111_ and TE^X^_121_ modes, ensures orthogonal polarization between adjacent resonators, minimizing the overlap of electric field components and inherently reducing mutual coupling. Second, the spatial placement of the dielectric resonators is carefully optimized to maintain adequate physical separation between ports, which reduces near-field interactions and contributes further to isolation. Finally, the incorporation of a full ground plane reflects unwanted signal components, preventing cross-coupling between resonators.

The proposed design is a combination of spatial diversity, polarization diversity, and a full ground plane to naturally achieve high isolation without relying on additional decoupling techniques. These design choices ensure a robust and efficient MIMO antenna suitable for sub-6 GHz 5G applications. This design eliminates the need for additional decoupling structures, simplifying the antenna design while maintaining high performance. Unlike non-DRA designs, where isolation typically requires external decoupling networks such as electromagnetic bandgap (EBG) structures, defected ground structures (DGS), or neutralization lines, the proposed DRA-based design leverages the material properties of the DRA and the strategic arrangement of antenna elements to achieve strong isolation. This approach not only eliminates the need for external decoupling networks but also ensures efficient and robust performance for MIMO.

## Antenna sensitivity evaluation

The presented antenna has a range of physical parameters that can be altered to see the antenna’s sensitivity. The variation in these parameters allows us to examine the optimization process and how it was tailored to reach the desired frequency bands. By altering these physical parameters, there will be numerous alterations in antenna radiation parameters, viz. frequency, polarization, radiation pattern, cross-pol isolation, and gain.

**Fig. 5:**
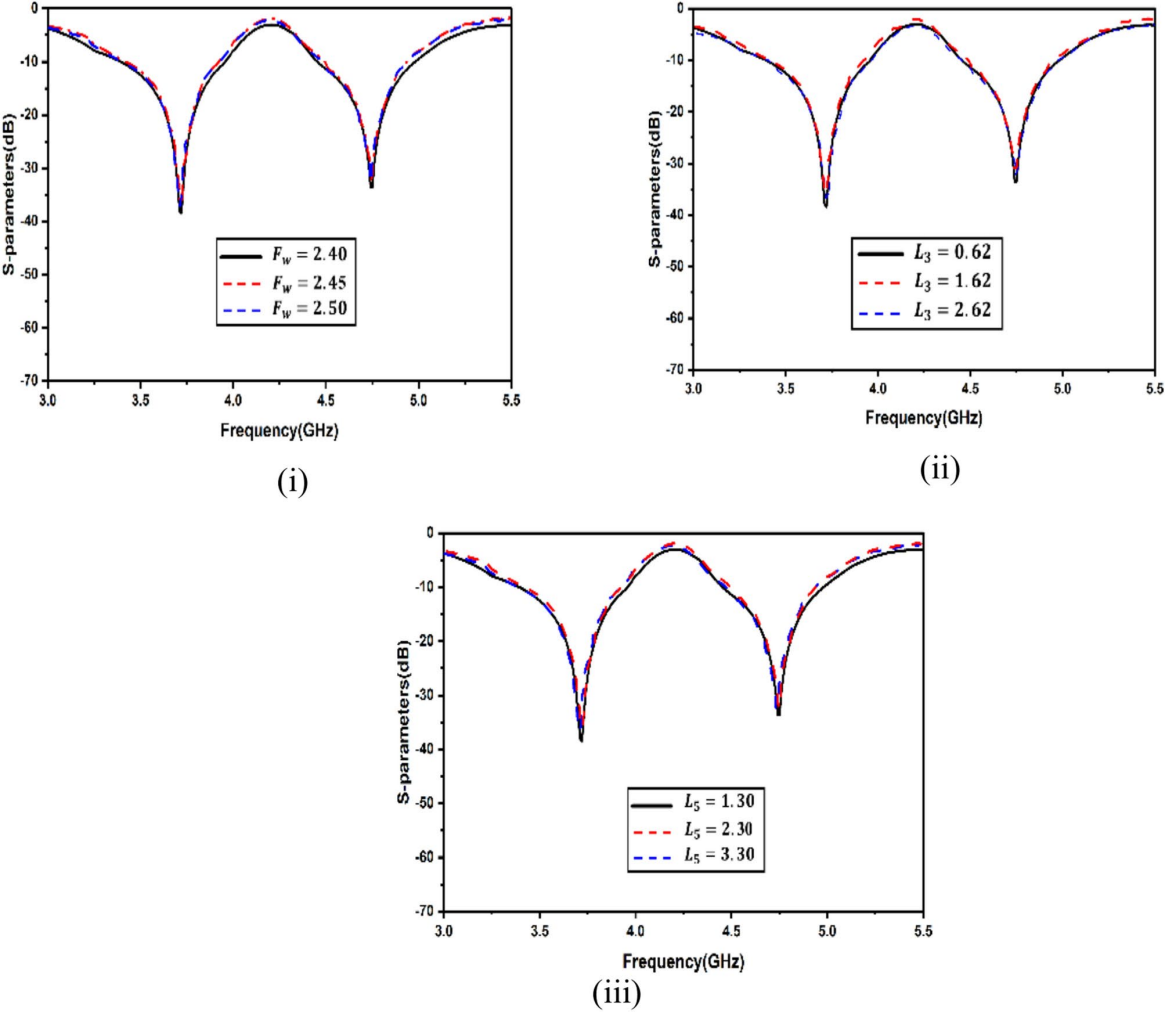
Single antenna analysis (**i**) feed width (**ii**) split gap (**iii**) diameter of outer line.

**Fig. 6:**
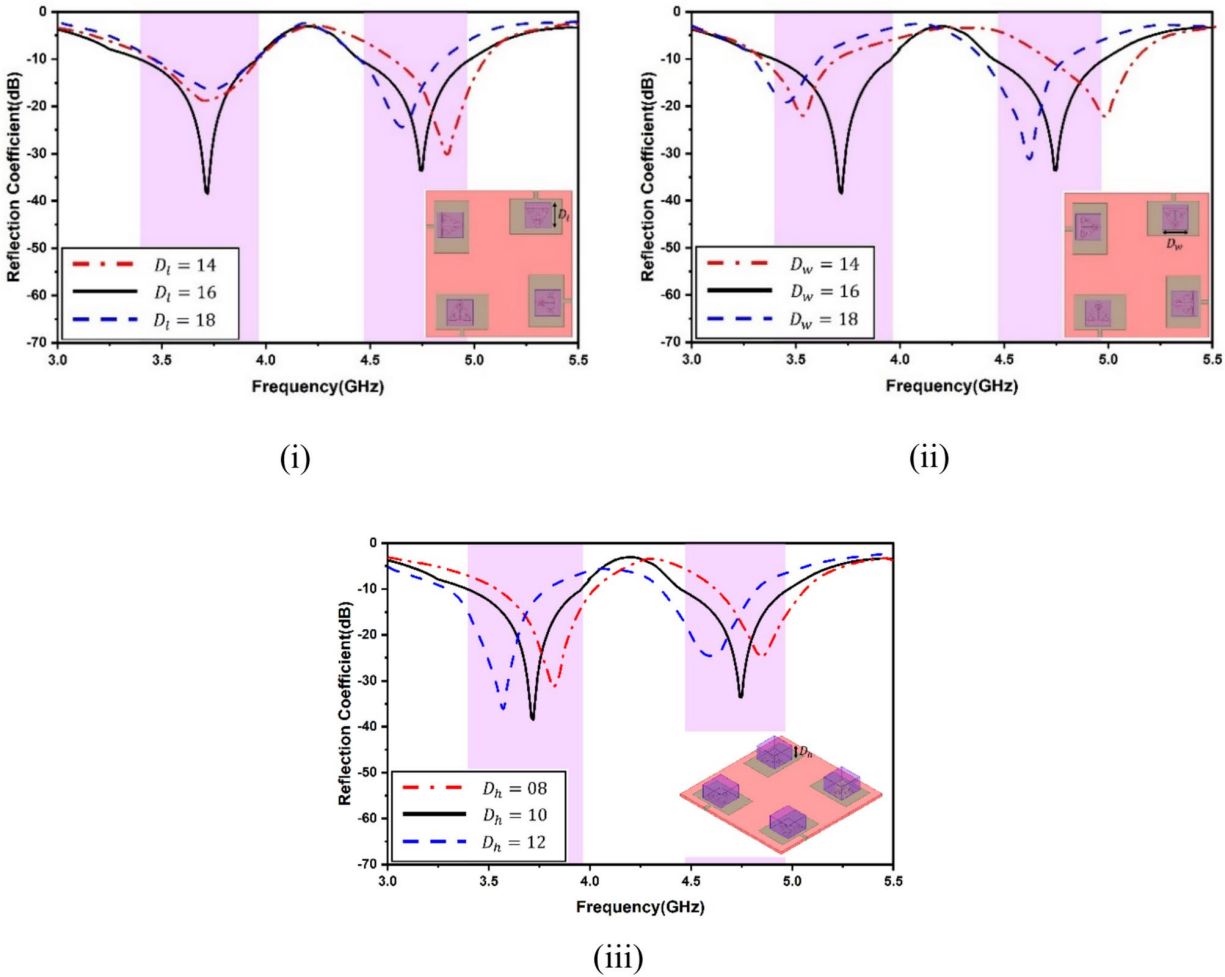
Antenna sensitivity to DR (**i**) DRA length (**ii**) DRA width (**iii**) DRA height.

**Fig. 7:**
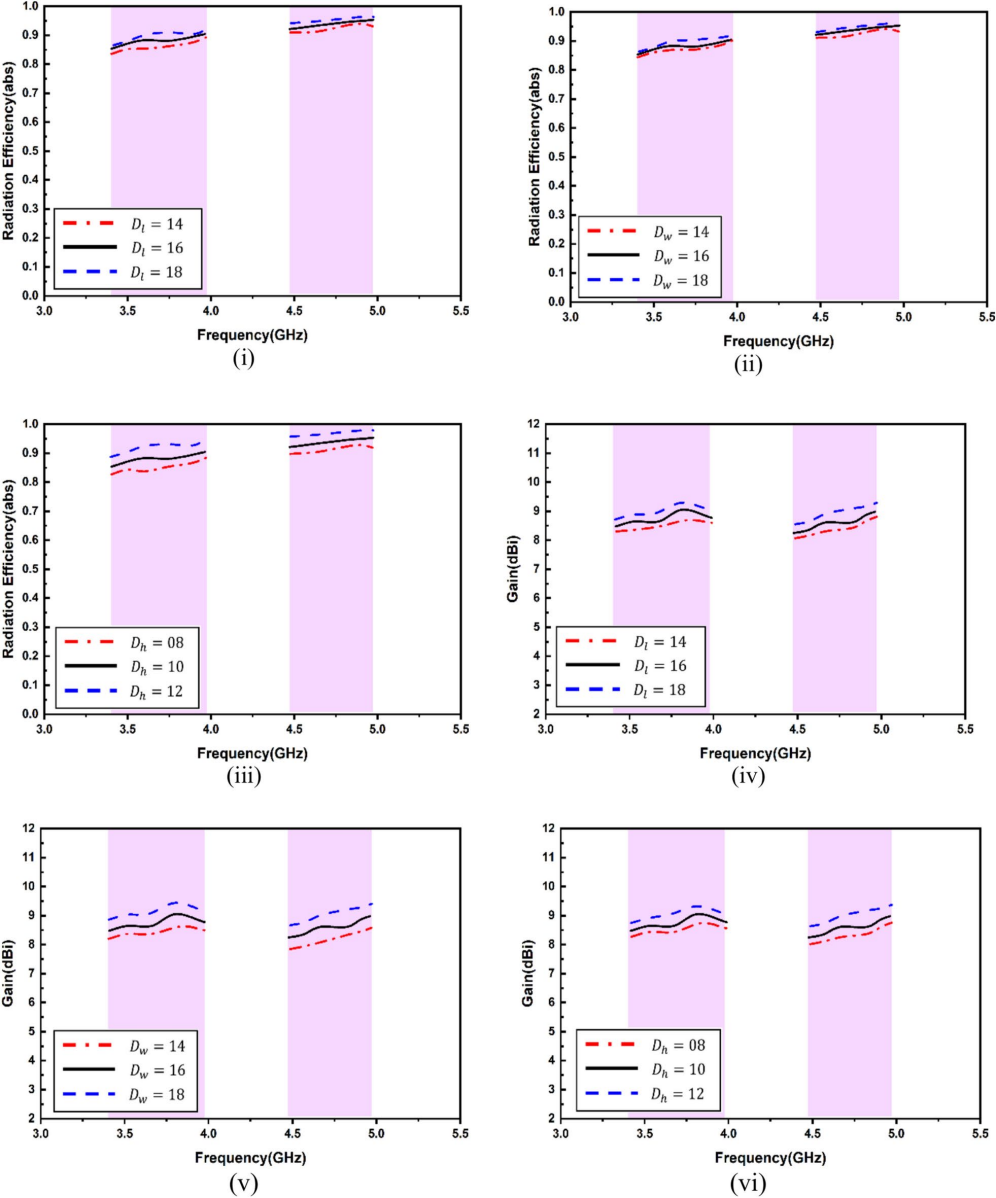
Antenna sensitivity analysis (**i**)–(**iii**) Efficiency, (**iv**)–(**vi**) Gain.

A range of simulations were carried out in the full-wave software simulator to reach the targeted frequency and optimize antenna parameters. The single antenna parametric analysis is shown in Fig. 5. The variation in feed width, split gap and slot width are exhibited. It is apparent that feed width is going to affect the matching, and hence, the reflection coefficient shall be sacrificed. The split gap and slot width intend to slightly vary the electrical length, causing minor shifts in the resonance. To exhibit the antenna sensitivity, selected variations in the DR were considered, as shown in Fig. 6. This is because tailoring of the DR electrical dimensions significantly alters the coupling of the electromagnetic field and, hence, the resonance. As apparent from Fig. 6(i), the second resonance gets altered with modifications in the length of the DR. The variations of the length were kept, and the electromagnetic coupling from the slots and their dimensions was realized. Figure 6(ii) shows that, again, the second frequency gets substantially affected compared to the first while varying the width. The height variations in Fig. 6(iii) exhibit significant alterations in both bands. The antenna shows significant sensitivity to the DR height and it becomes vital to select this height. The increase in DR height may enhance the radiation parameters of the antenna owing to the fact that dielectric material shall store a good quantum of the field within it due to its electrical properties, especially the effective dk of the antenna. It is pertinent to note that very high values of DR may not be feasible in packaging within communication devices. The sensitivity of antenna efficiency with respect to DR parameters has been shown in Fig. 7(i)–(iii), and the sensitivity of antenna gain with respect to DR parameters have been shown in Fig. 7(iv)–(vi).

It is evident that both antenna efficiency and gain are highly sensitive to the variation in the dimensions of the DR. However, it is observed that the variation in the dimensions of the DR also affects the antenna resonance. Hence, the optimized DR dimensions were utilized in the proposed design. Again, it is tremendously difficult to establish a relationship between the changing of the DR electrical dimensions against the antenna resonance as the electromagnetic coupling will vary significantly by slight modifications of the DR dimensions. This also stands true for the length and width of the DR.

## Results and discussions

**Fig. 8:**
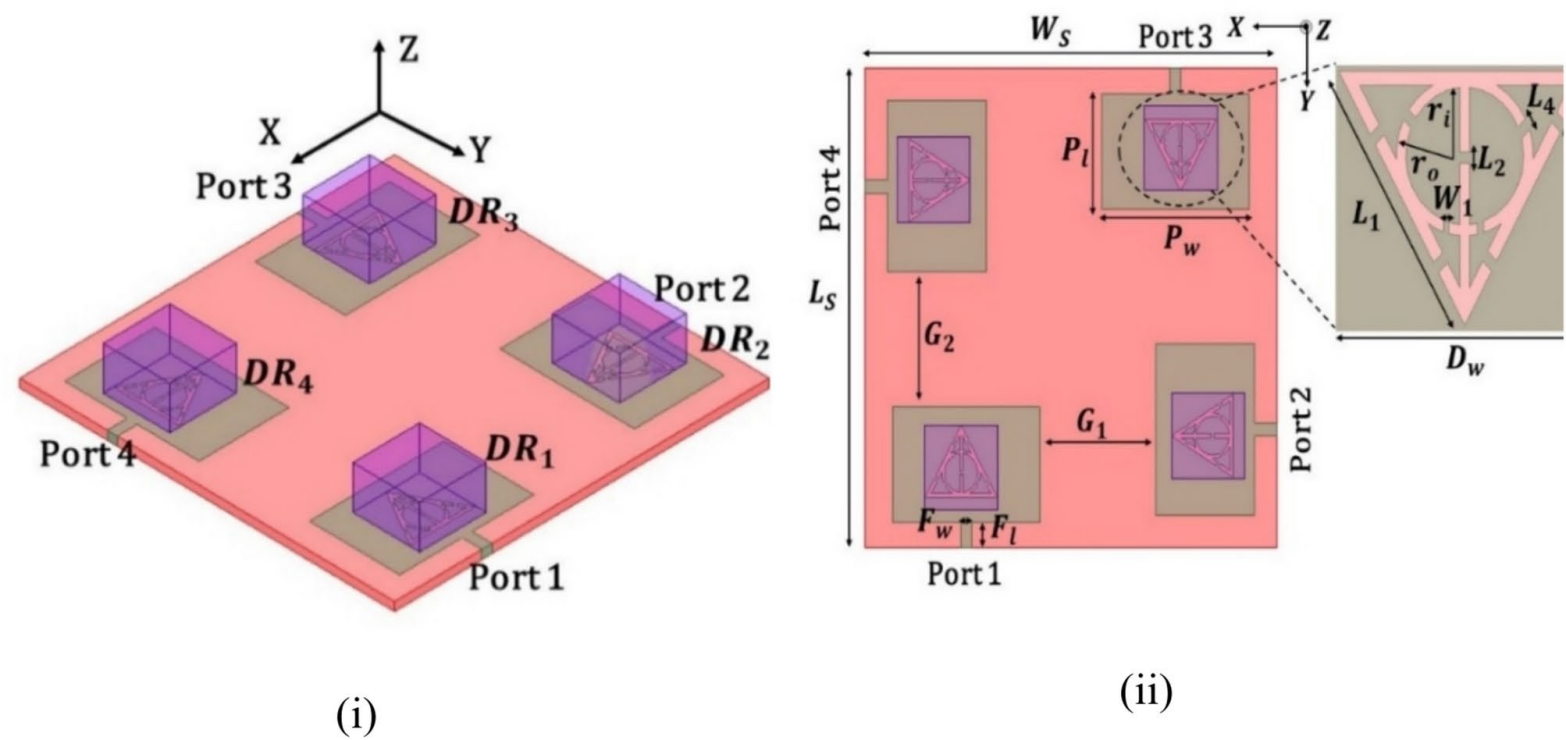
MIMO antenna (**i**) 3D (**ii**) Top view.

The designed MIMO antenna is illustrated in Fig. 8. The full-wave simulation software High Frequency Structure Simulator (HFSS) v22 was utilized for the simulation of the proposed antenna. The quad-ports are exiting the four DRs spatially kept apart. It is appropriate and intuitive that the higher the separation between elements, the lesser the inter-element coupling. The inter-element coupling is a key factor in achieving the diversity parameters of the MIMO antenna. This, however, comes with the tradeoff of a larger antenna footprint. To accommodate the distant DRs for the MIMO antenna structure, the substrate dimensions were enlarged, considering the inter-element coupling factor. Numerous software simulations were carried out to reduce the gap between DRs without sacrificing the isolation to keep the footprint electrically compact. The patch and DRs were arranged orthogonally on the antenna substrate to attain polarization diversity. The tailored MIMO antenna’s substrate dimensions were 90 mm in length and breadth. To achieve spatial diversity, the element distance was kept sufficiently far enough. The electrical dimension values of G1 and G2 are 0.314λ.

**Fig. 9:**
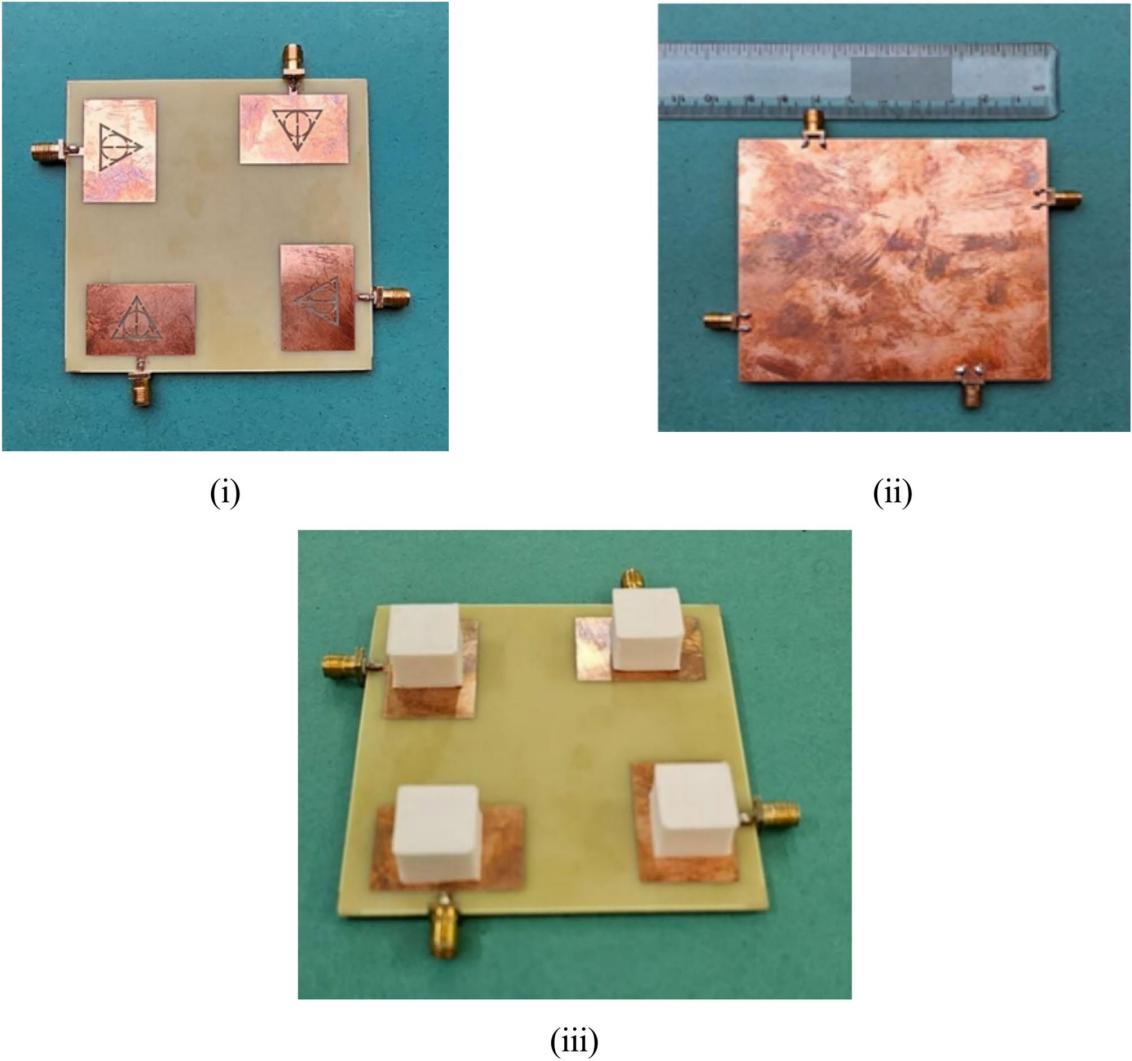
Fabricated prototype (**i**) without DR (**ii**) Ground (**iii**) with DR.

**Fig. 10:**
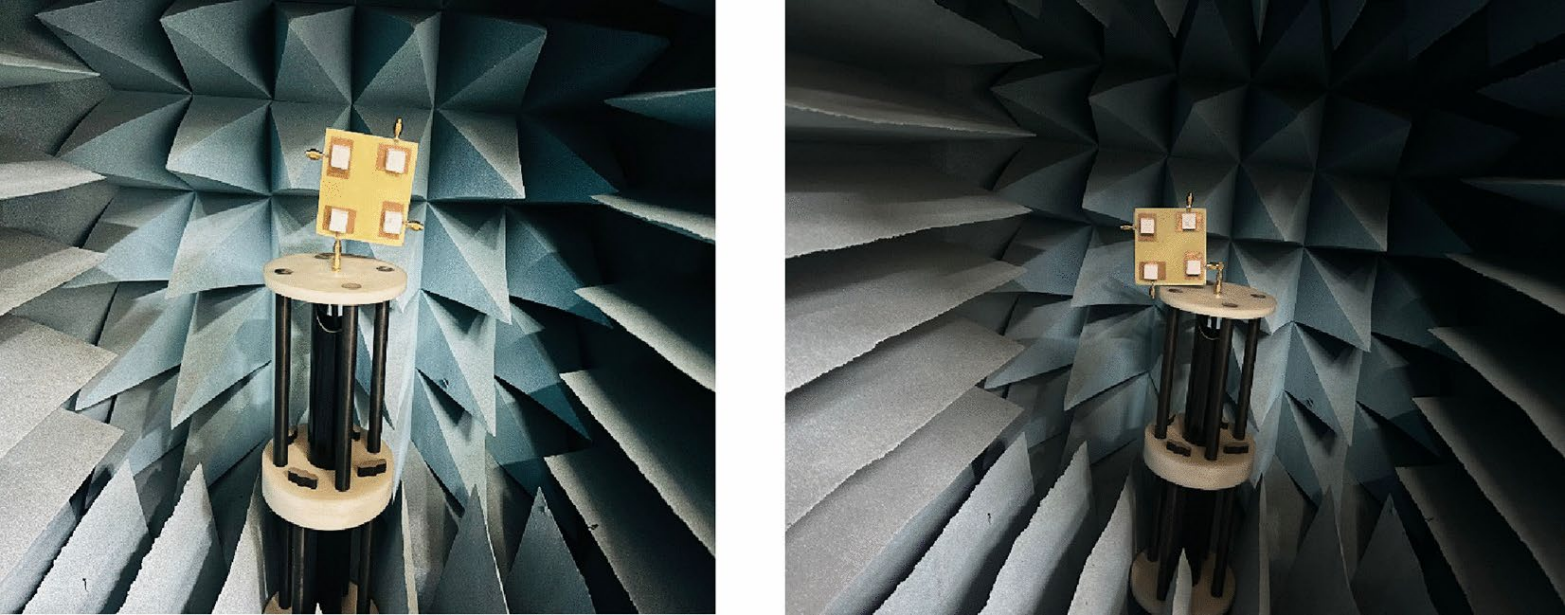
Experimental setup for antenna testing in anechoic chamber.

**Fig. 11:**
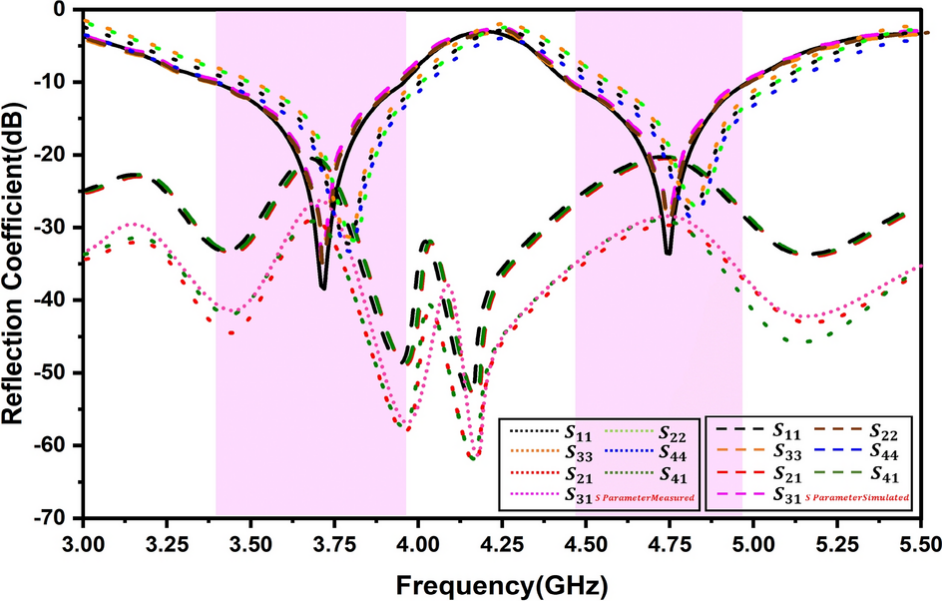
S-parameter for MIMO antenna simulated Vs measured.

The inter-element spacing between the antenna elements significantly affects the isolation. When the antenna elements are kept close to each other the mutual coupling between them increases substantially causing coupling to increase due to field interactions between the elements. The closer spacing can also create interference signals being transmitted or received by the antenna deterring the MIMO antenna performance. The larger spacing, however, shall reduce the isolation further but it shall affect the spatial diversity. Typically, the elements are separated by half or full wavelength to achieve a good level of isolation based on the targeted frequency. The attachment of the DRs on the patch resonator was carried out using a thin conducting paste. Figure 9 demonstrates the antenna prototype fabricated on FR-4 laminate. The laser-cut dielectric material was utilized considering the mechanical dimensions after model optimization. The manufactured sample was tested using N9912A VNA for measurement of scattering parameters. Figure 10 shows illustrates the anechoic chamber used for radiation pattern and efficiency testing. The dual-band resonance was achieved at 3.72 GHz and 4.75 GHz. The simulated Vs measured spectra are illustrated in Fig. 11 exhibiting a close relationship. There is a certain deviation in the simulate and measured results. These mismatch is due to mechanical fabrication issues or measurement device inaccuracies. The scattering parameters at a given port are measured by inducting termination at another port. These are typically 50 Ω matched terminations. The fractional bandwidths at the targeted frequencies are 14.7% and 11.57%, achieving better than 2:1 VSWR and meeting the target application requirements. Optimization techniques, such as multi-layer dielectric resonator (DR) structures, offer a promising method to further increase the antenna’s bandwidth, potentially achieving bandwidth improvements of 5% or more. However, while multi-layer designs can maintain a compact surface footprint, they may introduce fabrication challenges and increase the total volume, potentially impacting size-constrained applications. Despite these challenges, the proposed design could benefit from further optimization through multi-layer configurations, which could enable wider bandwidth, improved efficiency, and better isolation while remaining compact enough for practical use.

**Fig. 12:**
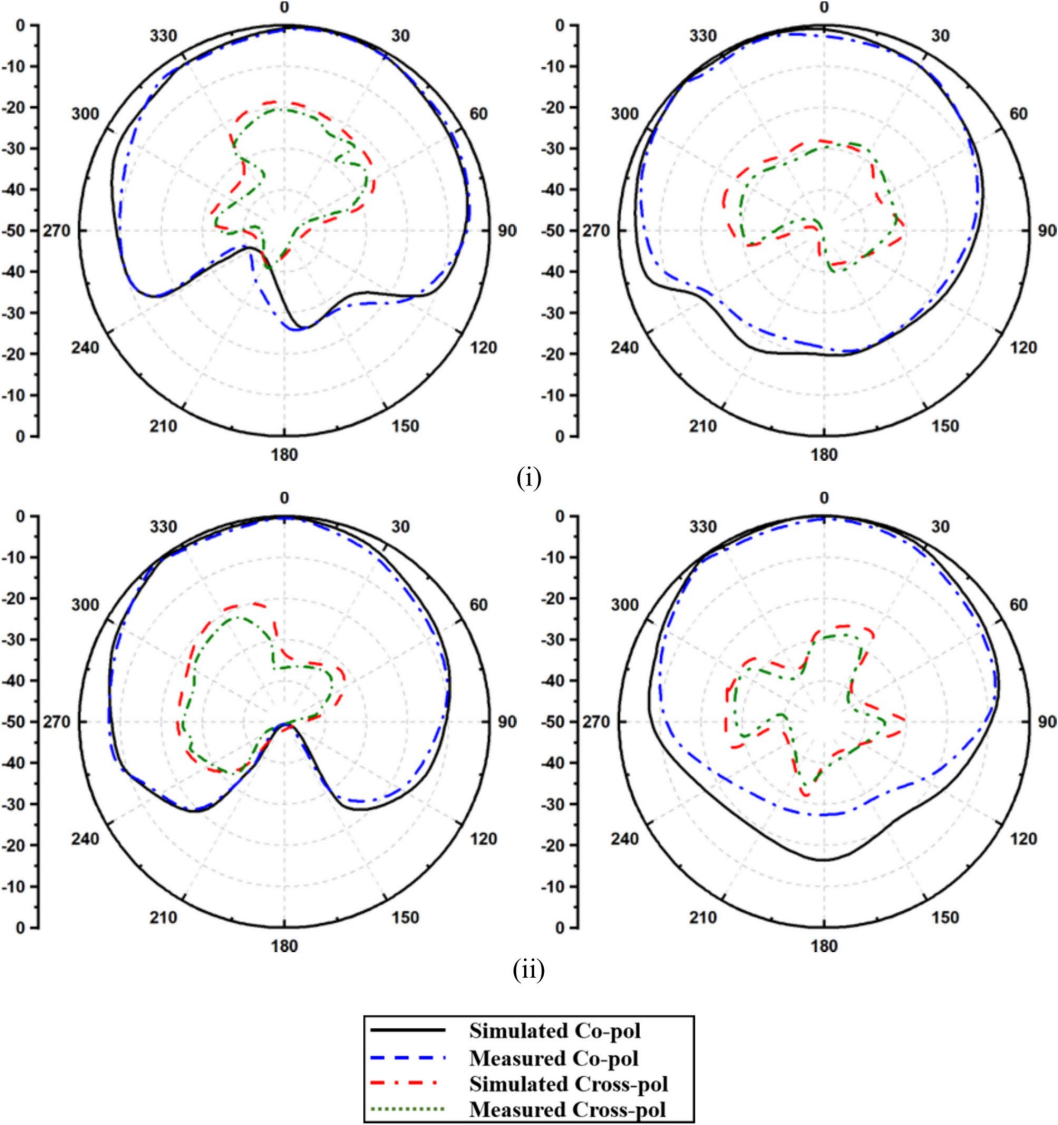
Radiation patterns at (**i**) 3.72 GHz (**ii**) 4.75 GHz.

The reflection coefficient plots on port 1 & port 2 are symmetrical and only one port results are shown for brevity. Owing to the design and orientation of the MIMO elements, high isolation was achieved. The elements were oriented orthogonally causing minimal electromagnetic coupling. The orthogonal alignment was crucial in attaining the polarization diversity. The significant separation between the parts results in port isolation measurements of 20.38 dB at 3.72 GHz and 21.86 dB at 4.75 GHz. Although the proposed antenna lacks an isolation method for enhancement, it delivers adequate isolation to achieve MIMO diversity parameters. Figure 12 depicts radiation patterns. One directional radiation pattern in the E-Field was observed due to the full ground plane profile, and intuitively, the H-plane provides an omnidirectional radiation pattern. The antenna gives an efficiency of 88.9% and 93.8% and a gain of 9.12 dBi and 8.58 dBi, respectively. The radiation efficiency was determined by measuring the total radiated power and accepted power, while total efficiency also accounts for mismatch losses.

To determine the gain of the Antenna Under Test (AUT), the Gain Comparison Method was utilized within an anechoic chamber, owing to its simplicity, reliability, and widespread industry adoption.

The procedure involves placing the Antenna Under Test (AUT) and a reference antenna inside an anechoic chamber at a fixed distance, ensuring a controlled environment. A Vector Network Analyzer (VNA) with a signal generator is used for power measurements. The received power (P_ref) is first recorded for the reference antenna across a selected frequency range. Then, the reference antenna is replaced with the AUT, and the corresponding received power (P_AUT) is measured at the same frequencies. The gain of the AUT is then calculated using the formula.

$${G}_{AUT}\left(f\right)= {G}_{ref}\left(f\right)+10\text{log}\left(\frac{{P}_{AUT}\left(f\right)}{{P}_{ref}\left(f\right)}\right)$$

By repeating this calculation across multiple frequency points, a set of gain values is obtained. These values are then plotted against frequency to generate the Gain vs. Frequency curve. The method provides an accurate representation of the antenna’s frequency-dependent gain performance, making it a widely used technique in antenna characterization.

**Fig. 13:**
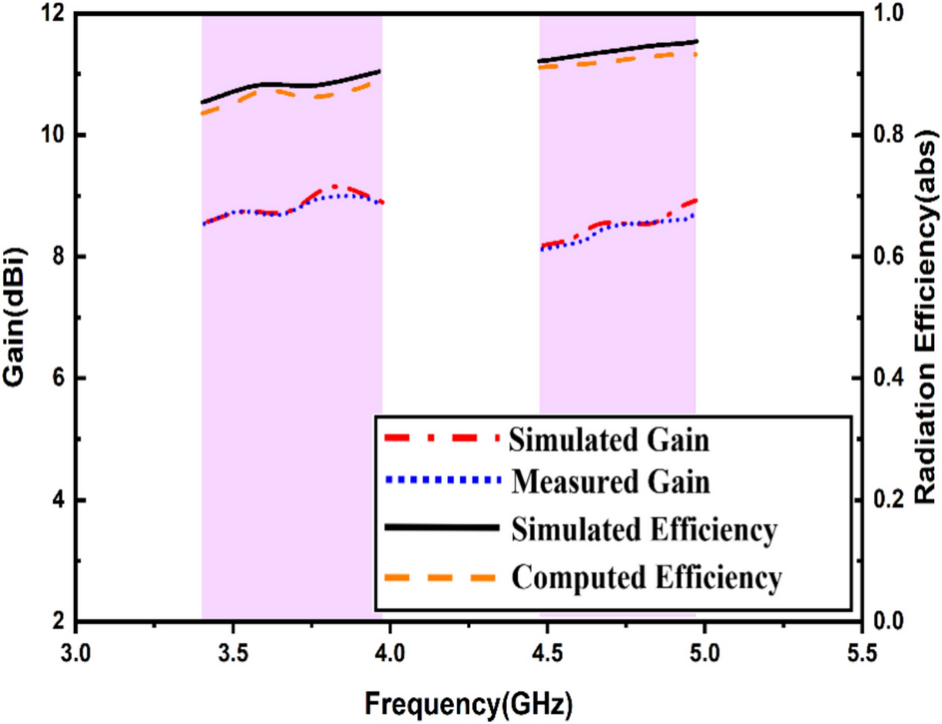
Gain and efficiency.

In the absence of the DR, the patch antenna only gives a peak gain of 5.67 dBi, whereas for the DR-based antenna, the gain increases to as high as 9.12 dBi and 8.58 dBi, as shown in Fig. 13. This improvement is attributed to the optimized excitation, spatial diversity, and the low conduction losses of the dielectric resonator, which collectively enhance gain and efficiency.

## MIMO diversity parameters

Wireless communication provides a hostile environment. MIMO communication increases spectral efficiency through diversity techniques. The diversity improves the communication reliability. The information chunk of the same information shall be sent over multiple independent channels, and due to inconsistent fading over these channels, the communication efficiency shall be enhanced. Multiple antennas in MIMO configurations are apt for attaining such spatial diversity.

**Fig. 14:**
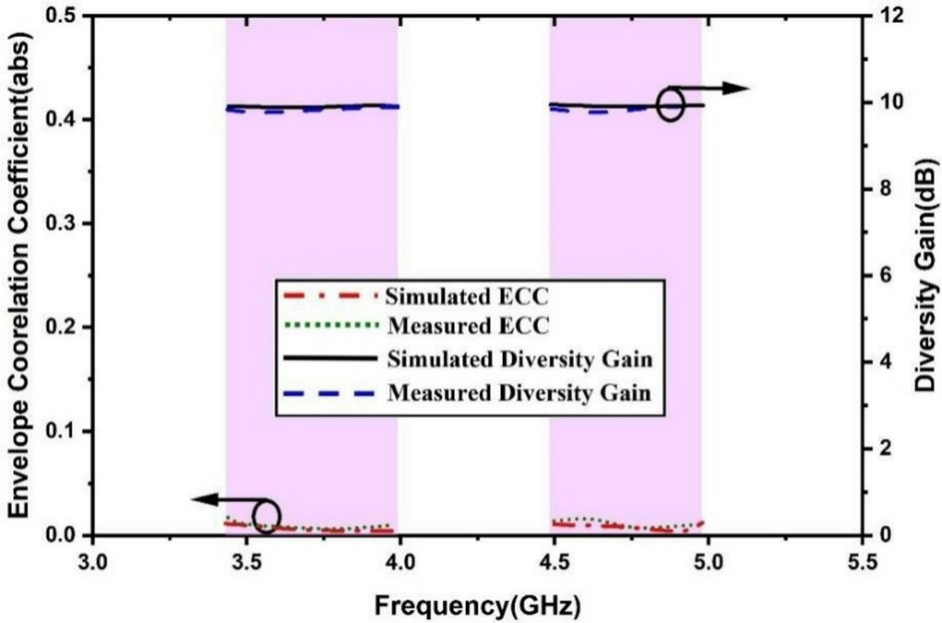
MIMO ECC and DG.

The envelope correlation coefficient (ECC) and diversity gain (DG) are crucial factors in the MIMO antenna system. The high envelope correlation causes reduced diversity gain as the diversity benefit is not fully realized. For instance, if the antenna elements are closely placed then the received signal may go exactly similar fading causing a poor diversity outcome. On the contrary, if the signals are independent then the MIMO system shall be able to achieve high diversity gain. To achieve the lower values of ECC, the antenna should be optimally spatially separated in a way that can maximize the diversity gain of the antenna. This shall help in achieving system reliability and maximum capacity. The ECC quantifies the correlations of radiation patterns. That horizontal polarization pattern cannot coincide with the vertical polarization pattern, resulting in a correlation of zero, as the radiation is designed to propagate in the other direction from the MIMO elements. The ECC between two adjacent elements in an n-element MIMO antenna can be determined via a far-field pattern as described in^20^:

4 $${\rho }_{e}=ECC=\frac{{\iint }_{0}^{4\pi }\left[{F}_{1}\left(\theta ,\varnothing \right)*{F}_{2}\left(\theta ,\varnothing \right)\right]d\tau }{{\iint }_{0}^{4\pi }|{F}_{1}\left(\theta ,\varnothing \right)|d\tau {\iint }_{0}^{4\pi }|{F}_{2}\left(\theta ,\varnothing \right)|d\tau }$$

where *R*_*i*_*(θ, φ)* and *R*_*j*_*(θ, φ)* are 3D patterns for two consecutive resonators *i* and *j*. The solid angle is *ω*, the Hermitian product is *, *θ* is the elevation angle, and *φ* is the azimuth angle. The expected values of ECC are typically less than 0.5 as exhibited in Fig. 14. The designed antenna has quite a suitable range of ECC. The Fig. 14 also depicts the directivity gain (DG). MIMO communication through spatial diversity resolves multipath signals providing a diversity gain. It substantially improves the intelligence of the system by recovering the data from multiple independent channels undergoing fading due to unexpected channel characteristics. The DG can be calculated by^21^:

5 $$DG=10 X(1-\left|{ECC}^{2}\right|)$$

The selection of the TE^X^_111_ and TE^X^_121_ modes in the proposed design offers several distinct advantages, making them particularly suitable for MIMO applications. These modes ensure broadside radiation patterns, which are highly desirable for achieving uniform coverage in MIMO systems. Additionally, their excitation enables dual-band functionality, as demonstrated by the resonances at 3.72 GHz and 4.75 GHz. The TE^x^_111_ and TE^X^_121_ modes also exhibit high efficiency, with values of 88.9% and 93.8%, respectively, and significant peak gain of 9.12 dBi and 8.58 dBi, making them ideal for sub-6 GHz wireless communication applications. Another significant advantage is the polarization diversity achieved through the orthogonal nature of these modes, which minimizes mutual coupling and enhances the reliability of MIMO systems by mitigating multipath fading effects^47^.

When compared to other modes, higher-order modes such as TE^x^_211_ or TE^y^_311_ may offer additional resonances, but they often introduce trade-offs, including increased design complexity, higher mutual coupling, and reduced radiation efficiency. In contrast, fundamental modes like TE^x^_111_ and TE^X^_121_ are optimal for compact designs as they inherently maintain low loss and high isolation due to their mode structure. These advantages, including polarization diversity, make the chosen modes well-suited for practical and efficient MIMO antenna implementations^48^.

The mean effective gain (MEG) is one of the crucial parameters for interpreting the MIMO performance. Given the multiple channel conditions, MEG provides the ratio of received power in reference to the isotropic antenna. The MEG can be given by^21^:

6 $${MEG}_{i}=0.5\left[1-\sum_{j=1}^{N}{\left|{S}_{ij}\right|}^{2}\right]$$

**Fig. 15:**
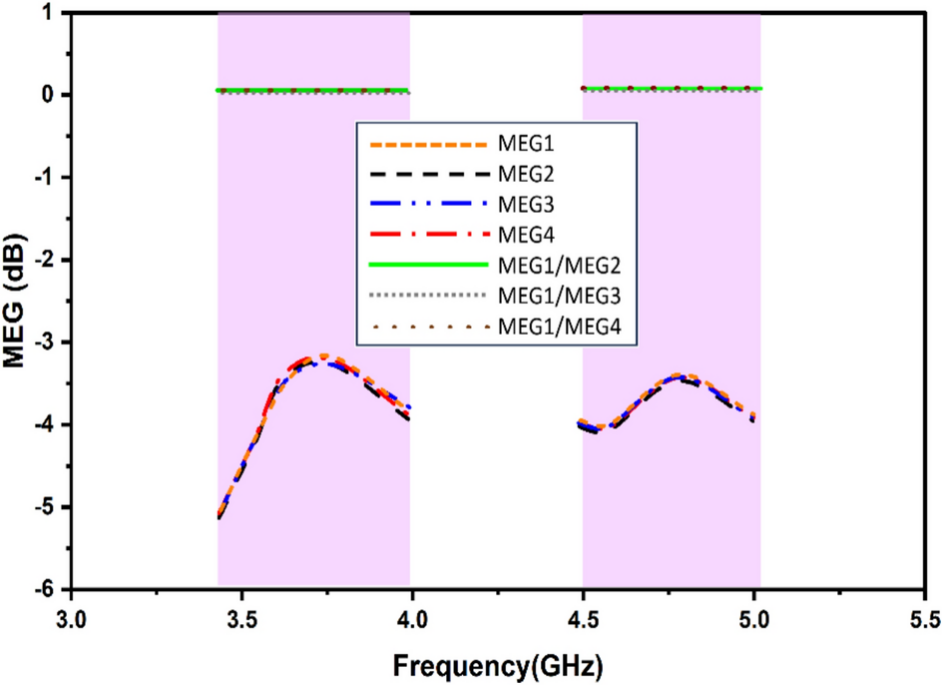
MIMO MEG.

Due to the sparse placement and lower mutual coupling between the antenna elements, the power ratio is extremely good. Figure 15 illustrates the MEG power ratios for the four-port antenna configuration.

**Fig. 16:**
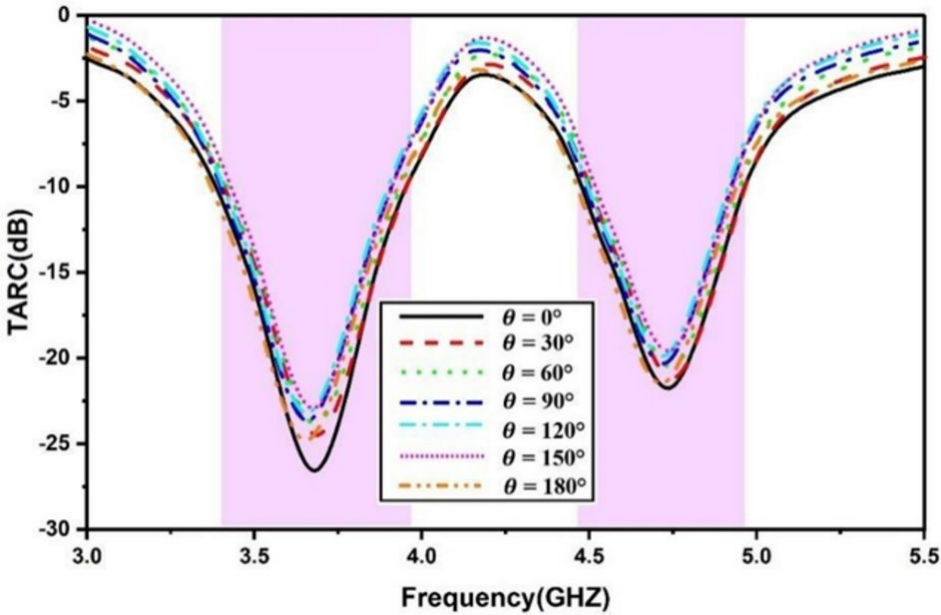
MIMO TARC.

**Fig. 17:**
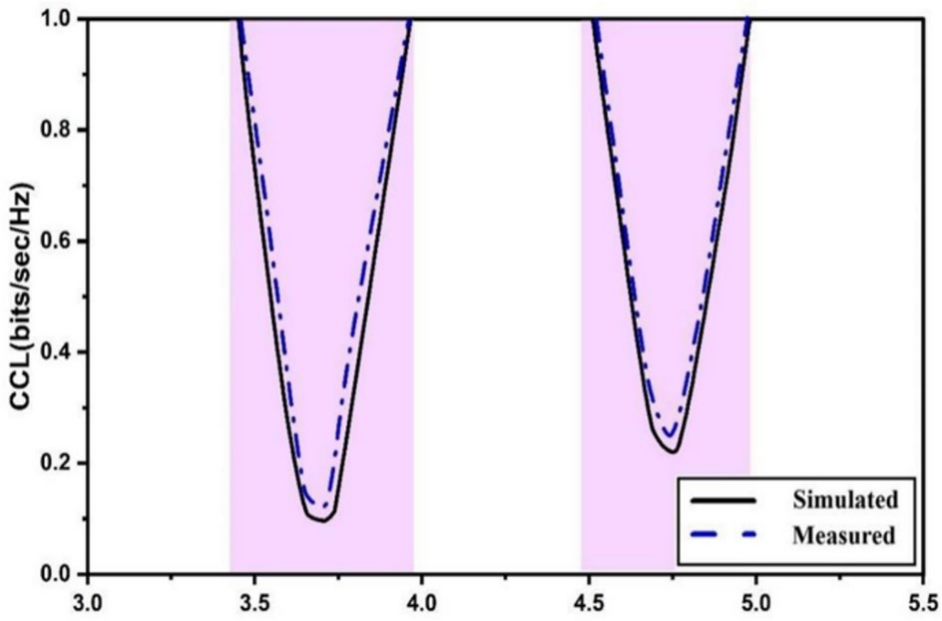
MIMO CCL.

The TARC gives the reflected-to-incident power ratio for the n-port system. Figure 16 illustrates the TARC of the proposed quad-port antenna which can be calculated using Eqs. (7 and 8)^21^. The obtained TARC is better than -22 dB for the given antenna. The inter-element correlation is a prime factor for diversity in the antenna. The channel capacity is bound to increase with incremental numbers of antenna elements closing near to maximum channel capacity. Primarily, it provides the measure of maximum bound of information transmission without incurring any losses in multipath communications. The increase in the number of resonating elements is restricted by the antenna form factor along with the coupling and hence it needs to be refrained to a certain number. Equations (9–11) provide CCL computation^21^. The desirable value of CCL is about 0.6 bits/s/Hz, and the proposed design is quite capable of providing necessary CCL values as presented in Fig. 17. The comparison of various antenna parameters of the MIMO antenna is tabulated in Table 2. It is evident that the presented DR-based antenna suits the target application requirements.

7 $$TARC=\frac{\sqrt{{\sum }_{n=1}^{N}{\left|{b}_{n}\right|}^{2}}}{\sqrt{{\sum }_{n=1}^{N}{\left|{a}_{n}\right|}^{2}}}$$

8 $${b}_{n}=\left[S\right]{a}_{n}$$

9 $$CCL=-{log}_{2} det\left({\Psi }^{R}\right)$$

10 $$\Psi^{R} = \left[ {\begin{array}{*{20}c} {\rho_{11} } & {\rho_{12} } & {\rho_{13} } & {\rho_{14} } \\ {\rho_{21} } & {\rho_{22} } & {\rho_{23} } & {\rho_{24} } \\ {\rho_{31} } & {\rho_{32} } & {\rho_{33} } & {\rho_{34} } \\ {\rho_{41} } & {\rho_{42} } & {\rho_{43} } & {\rho_{44} } \\ \end{array} } \right]$$

11 $$\rho_{ii} = 1 - \sum\limits_{n = 1}^{4} {\left( {S_{in}^{*} S_{ni} } \right)} ;\;\rho_{ij} = - \sum\limits_{n = 1}^{4} {\left( {S_{in}^{*} S_{nj} } \right)} \;\; {\text{For}}\;i,j = {1},{2},{3},\;{\text{or}}\;{ 4}$$

**Table 2:** Antenna comparison with existing literature.

References	Frequency (GHz)	Gain (dBi)	Bandwidth (%)	Isolation (dB)	Efficiency (%)	ECC	CCL bits/s/Hz	DG (dB)	TARC (dB)
^21^	4.5, 5	7.67, 8.32	2.64, 1.2	20	88.0	0.037	0.2	9.99	− 18.8
					86.3				7
^34^	3.21–3.81	3.64	17	10	90	< 0.02	–	9.9	20
^35^	3.72–3.82, 4.65–4.76, 6.16–6.46	2.5	2.6, 2.3, 4.75	16	89	< 0.1	0.2	9.8	− 10
^36^	3.20–5.8	3.5	58.56	17.5	85	< 0.01	0.4	9.8	–
^37^	4.9–5.725	4.8	28.8	15.4	70	< 0.15	–	9.8	–5
^38^	5.2–5.7, 11.8–17.3	3.05, 5.27	9, 3.61	20	90	< 0.007	0.4	9.96	− 10
^39^	5.29–6.12, 26–29.5	5.13, 5.6	15, 13	20	73	< 0.04	< 0.4	9.9	–
^40^	2.37–5.85	4.0	84.12	17.5	85	< 0.05	–	9.8	− 10
^19^	5.52–6.2 GHz	5.12	10	15	91.5	0.050	0.40	7.5	–
^41^	3.3–4.2 GHz	8.8	8–15	15	92	0.045	0.35	8	− 21
^42^	3.3 GHz L-3.9 GHz	5.8, 6.2	6	20	88.6,90	0.01	0.1	9.9	-22.46
^43^	3.4–3.8 GHz	8	11.76%	> 22	90.2	0.6	0.5	7.2	− 19.8
Proposed design	3.72, 4.75	9.12, 8.58	14.7, 11.57	20.38 21.86	88.9	< 0.04	< 0.6	9.9	− 22
					93.8				

The performance of the proposed MIMO dielectric resonator antenna (DRA) is Explained by comparing its ECC and CCL with state-of-the-art designs. Table 2 provides a comparison of key metrics, showcasing the efficiency gains of the presented design. However, authors have highlighted the practical advantages of the proposed antenna in achieving superior ECC and CCL.

### ECC comparison

Envelope correlation coefficient (ECC) is critical for MIMO performance, as it measures the independence of radiation patterns and thus the diversity gain. The proposed design achieves an ECC of 0.042, which is significantly below the commonly accepted threshold of 0.5, demonstrating its suitability for high-performance 5G systems. This result is competitive with or superior to contemporary designs such as CPW-fed Quad-Port DRA^19^, which achieved an ECC of 0.05. Metasurface-Based MIMO Antenna^25^, with ECC values around 0.06. The lower ECC in the presented antenna is attributed to Strategic placement of dielectric resonators, maximizing spatial diversity. Full-ground plane architecture, which minimizes inter-port coupling.

### CCL comparison

Channel capacity loss (CCL) directly impacts the efficiency of data transmission in MIMO systems. The proposed antenna exhibits a CCL of less than 0.2 bits/s/Hz, which exceeds the performance of designs such as Multilayer MIMO Systems (18), reporting CCL values nearing 0.4 bits/s/Hz. Hybrid MIMO Antenna^23^, with CCL between 0.3 and 0.5 bits/s/Hz.

The evaluation of MIMO parameters further validates the antenna’s suitability for 5G Sub-6 GHz applications. The Envelope Correlation Coefficient (ECC) of 0.042 ensures minimal inter-element correlation, confirming high spatial diversity. A Diversity Gain (DG) of 9.99 enhances system reliability by mitigating multipath fading. The Channel Capacity Loss (CCL) below 0.2 bits/s/Hz ensures efficient data transmission, while a Total Active Reflection Coefficient (TARC) better than −22 dB indicates optimal impedance matching. Additionally, the Mean Effective Gain (MEG) analysis confirms the antenna’s robustness in varying propagation environments. These findings collectively demonstrate the effectiveness of the proposed design in achieving high data rates and reliable performance in next-generation wireless communication systems.

## Challenges and future trends

Scaling the design to larger MIMO arrays, such as 8 × 8 or beyond, presents several challenges that need to be addressed for effective implementation. One major challenge is mutual coupling; as the number of elements increases, maintaining sufficient physical separation between them becomes more difficult, which can lead to higher coupling and degraded performance. Additionally, the complexity of the feed network grows significantly, as ensuring uniform power distribution and minimal phase error across all elements becomes increasingly challenging. Another issue is size constraints; the overall size of the array increases with the number of elements, potentially limiting its application in compact devices. Furthermore, fabrication tolerance poses a concern, as the alignment and precise positioning of additional resonators can result in inconsistencies in performance due to manufacturing limitations. To overcome these challenges, several solutions are proposed. Modular design can be adopted, where the array is divided into smaller, independent modules (e.g., 2 × 2 sub-arrays) with inherent isolation, which can then be combined to form larger arrays^44^. Hybrid techniques, such as integrating DRAs with planar antennas, can help achieve compact and scalable configurations^45^. Additionally, the use of advanced materials with higher permittivity allows for the reduction of resonator size without compromising performance^46^. By implementing these strategies, the design can be effectively scaled to larger arrays while maintaining its performance and reliability.

Achieving an electrically compact design with sufficient inter-element isolation and maintaining antenna bandwidth remains a significant challenge, requiring careful optimization of multiple parameters. Smaller antennas tend to have narrower bandwidths, but the use of DRAs can help maintain stable gain and bandwidth. However, keeping the DRA elements in close proximity can reduce isolation. A potential future approach involves employing multi-layer DRAs, which can reduce the antenna’s electrical size in terms of surface footprint while maintaining performance. Another innovative strategy could involve fractal designs in DRAs or the integration of metamaterials. Additionally, advanced signal processing algorithms could be utilized to balance the tradeoff between isolation and bandwidth, though these methods may increase system complexity. While achieving compactness can save space and reduce power consumption, it introduces design challenges for isolation, which can be mitigated by incorporating effective decoupling mechanisms.

## Conclusion

A quad-port MIMO dielectric resonator antenna (DRA) is designed for 5G Sub-6 GHz applications, operating at 3.72 GHz and 4.75 GHz with fractional bandwidths of 14.7% and 11.57%. The antenna achieves high isolation (20.38 dB & 21.86 dB) using spatial arrangement and orthogonal polarization, eliminating the need for external decoupling structures. It provides peak gains of 9.12 dBi & 8.58 dBi with radiation efficiencies of 88.9% & 93.8%. The Envelope Correlation Coefficient (ECC) of 0.042, Diversity Gain (9.99), and Channel Capacity Loss (CCL < 0.2 bits/s/Hz) confirm excellent MIMO performance. The antenna’s compact design, high efficiency, and polarization diversity make it suitable for high-data-rate 5G communication systems.

## Data Availability

The data used to support the findings of this study are included in the article.
